# Genomic Features and Construction of Streamlined Genome Chassis of Nisin Z Producer *Lactococcus lactis* N8

**DOI:** 10.3390/microorganisms10010047

**Published:** 2021-12-27

**Authors:** Wanjin Qiao, Fulu Liu, Xing Wan, Yu Qiao, Ran Li, Zhenzhou Wu, Per Erik Joakim Saris, Haijin Xu, Mingqiang Qiao

**Affiliations:** 1 Key Laboratory of Molecular Microbiology and Technology, Ministry of Education, College of Life Sciences, Nankai University, Tianjin 300071, China; qiaowanjin@outlook.com (W.Q.); 1120170379@mail.nankai.edu.cn (F.L.); qiaoyu0507@outlook.com (Y.Q.); 2 Department of Microbiology, Faculty of Agriculture and Forestry, University of Helsinki, 00014 Helsinki, Finland; xing.wan@helsinki.fi (X.W.); ran.li@helsinki.fi (R.L.); per.saris@helsinki.fi (P.E.J.S.); 3 Department of Bacteriology and Immunology, Human Microbiome Research Program, Faculty of Medicine, University of Helsinki, 00014 Helsinki, Finland; 4 State Key Laboratory of Medicinal Chemical Biology and Tianjin Key Laboratory of Protein Sciences, College of Life Sciences, Nankai University, Tianjin 300071, China; naturepower@nankai.edu.cn; 5 School of Life Sciences, Shanxi University, Taiyuan 030006, China

**Keywords:** *Lactococcus lactis*, genomic feature, plasmid, prophage, genomic island, NRPS–PKS, streamlined genome chassis

## Abstract

*Lactococcus lactis* is a commonly used fermenting bacteria in cheese, beverages and meat products. Due to the lack of simplified chassis strains, it has not been widely used in the fields of synthetic biology. Thus, the construction of lactic acid bacteria chassis strains becomes more and more important. In this study, we performed whole genome sequencing, annotation and analysis of *L. lactis* N8. Based on the genome analysis, we found that *L. lactis* N8 contains two large plasmids, and the function prediction of the plasmids shows that some regions are related to carbohydrate transport/metabolism, multi-stress resistance and amino acid uptake. *L. lactis* N8 contains a total of seven prophage-related fragments and twelve genomic islands. A gene cluster encoding a hybrid NRPS–PKS system that was found in *L. lactis* N8 reveals that the strain has the potential to synthesize novel secondary metabolites. Furthermore, we have constructed a simplified genome chassis of *L. lactis* N8 and achieved the largest amount of deletion of *L. lactis* so far. Taken together, the present study offers further insights into the function and potential role of *L. lactis* N8 as a model strain of lactic acid bacteria and lays the foundation for its application in the field of synthetic biology.

## 1. Introduction

*Lactococcus lactis*, a group of Gram-positive, catalase-negative, mesophilic fermentative bacteria producing lactic acid from sugar [1], has a long history of safe use in the fermented food industry and is granted a “GRAS” (generally regarded as safe) status. *L. lactis* has three subspecies, two of which are routinely employed in the dairy fermentation sector, i.e., subspecies (subsp.) *lactis* and subsp. *lactis biovar diacetylactis*, which distinguishes itself based on the citrate metabolism. The remaining is *L. lactis* subsp. *hordniae* isolated from the leafhopper *Hordnia circellata* [2]. Genetically, a typical *L. lactis* chromosome ranges in size from ~2.2 to 2.6 Mb and is often accompanied by plasmid complements [3] and multiple remnant prophages [4].

*L. lactis* N8 (N8) is a nisin Z producer isolated from milk in Finland, and the knowledge gained from fundamental research on this nisin production strain has been exploited for a wide variety of biotechnological applications. N8 is robust and genetically amenable, which has facilitated the analysis of introduced lactococcal and heterologous DNA. Due to the feature of considerable nisin yield, the research on this strain so far has mainly focused on its nisin synthetic gene cluster and increasing nisin yield [5,6]. The gene cluster for nisin synthetic (*nisZBTCIPRKFEG*) in strain N8 has been thoroughly studied in the past years. The NisB and NisC are responsible for the dehydration and cyclization of the precursor nisin [7]. The NisT is responsible for the transport of nisin and is also responsible for the dehydration and formation of lanthionine during the maturation of nisin, which occurs independently of the transport [5]. The NisI could have a supportive role in nisin immunity [8]. The NisP is essential to the maturation of nisin, and it has catalytic activity for nisin leader-peptide cleavage [9]. The NisRK, as a two-component regulatory system, has been characterized in other lactic acid bacteria [10]. The NisFEG, as a transporter complex, is important for the development of nisin immunity [8,11].

Researchers have tried to increase the nisin production of N8 through various methods, such as: (1) by improving the nisin immunity of the producer strain [12]; (2) by optimizing the culture conditions to achieve a high nisin yield of the producer strain [6]; (3) by using Mu transposition technology to build a library to screen genes that affect nisin production and nisin immunity [13,14].

Moreover, strain N8 has been used in food applications. For example, *L. lactis* N8 and *Saccharomyces boulardii* SAA655 were used in idli batter to increase the nutritional values of the food products by boosting the riboflavin and folate levels by 40 to 90% [15]. Recently, co-culturing *L. lactis* N8 with a bacterial cellulose-producing strain *Enterobacter* sp. FY-07 has successfully endowed bacterial cellulose with antibacterial properties and extended the application of N8 in food packaging [16]. Our group have heterologously secreted leucocin C in N8 and successfully achieved nisin and leucocin C co-expression. The recombinant bacteria exhibited highly efficient antimicrobial activity against *L. monocytogenes* [17].

In the past 30 years, a great deal of studies on N8 have been published, demonstrating its importance in the field of microbiology and biotechnology. However, the characteristics of its genome have not been deeply explored. Furthermore, the recent appraisal for the potential application of *L. lactis* strains in oral vaccine delivery may expand the importance of lactococcal investigations into the medical/pharmaceutical arena [18] as probiotic lactic acid bacteria are becoming more and more important in the field of synthetic biology [19].

In order to expand its application in the fields of the pharmaceutical arena, synthetic biology and metabolic engineering, in this study, we sequenced the whole genome of *L. lactis* N8. Through an in-depth analysis of the whole genome, we predicted the prophages and genomic islands of N8 that could be deleted to decrease the metabolic burden of the strain and increase the yield of useful substances. Through the analysis of the whole genome, we obtained the theoretical background, and, based on this, we carried out the construction of the N8 streamlined genome chassis using our established genome editing methods [20].

## 2. Materials and Methods

### 2.1. Strain and Media

All the strains used in this study were listed in Table 1. The original strain *L. lactis* N8 was isolated from milk in Finland [21]. M17 broth was purchased from Hope Bio-Technology (Qingdao, China). N8 and its derivatives were grown on GM17 agar plates (supplemented with 0.5% (*w/v*) glucose). After incubation overnight at 30 °C, a single colony was inoculated in GM17 and cultured overnight at 30 °C. Primers used in this study were purchased from GENEWIZ (Suzhou, China) and are listed in Supplementary Table S1.

### 2.2. DNA Extraction and Genome Sequencing

Genomic DNA (gDNA) was extracted from 2 mL overnight cultures of *L. lactis* N8 cells using MagAttract HMW DNA kit (Qiagen, Hilden, Germany) according to the manufacturer’s protocol. Isolated gDNA was subjected to whole genome sequencing performed by the DNA Sequencing and Genomics Laboratory (Helsinki Institute of Life Science, University of Helsinki, Helsinki, Finland). The high throughput sequencer Pacbio Sequel II System (Pacific Biosciences, San Francisco, CA, USA) was used for sequencing, and assembling was done by SMRT Link 9 Analysis software (Pacific Biosciences). Polished contigs were used for genome analysis. A more detailed explanation of the sequencing method can be found in the genome announcement of *L. lactis* subsp. *lactis* N8 [21].

### 2.3. Genome Analysis

#### 2.3.1. Genome Annotation

The annotation of the genome was performed using the NCBI Prokaryotic Genome Annotation Pipeline (PGAP), and the resultant annotated proteome was further annotated with Cluster of Orthologous Groups (COG) categories and KEGG pathways using the Prokka version 1.14.6 and eggNOG mapper [26,27,28]. Circos software version 0.69-9 was used to make circle maps of the genome and large plasmids after genome assembly and annotation. Comparison of the homology of two large plasmids was performed and visualized using EasyFig [29]. All *L. lactis* plasmid data were extracted from NCBI https://www.ncbi.nlm.nih.gov/genome/browse/#!/plasmids/156/ (accessed on 5 July 2021). To differentiate plasmids and chromosomal accessory genes and highlight the plasmid contribution, COG annotation was conducted utilizing WebMGA server8 [30], using as input protein files predicted by Prokka. The flower plot was made using an online platform (https://www.genescloud.cn/; accessed on 5 July 2021).

#### 2.3.2. Pan-Genome and Phylogenetic Analysis

To establish the accurate position of N8 within the population structure of *L. lactis*, we reconstructed the phylogeny using all available 202 *L. lactis* strains, including subsp. *lactis*, subsp. *lactis bv. diacetylactis* and subsp. *hordniae* (data retrieved by latest 5th July 2021, Supplementary Table S2). To ensure the assembly quality of dataset, only genomes with an N50 size over 20 kbp were selected. The pangenomes of *L. lactis* were inferred using Roary version 3.11.2 [31]. First, the nucleotide fasta files from all selected strains were downloaded from NCBI together with the GFF files and converted to GFF3 using a script (Supplementary Table S3). Regarding the strains for which GFF3 files were not provided by NCBI or could not pass the preliminary analysis by Roary, their nucleotide fasta files were downloaded and annotated using Prokka to obtain a compatible GFF3 file [26]. Second, the GFF3 annotations were provided to Roary to calculate the pangenome of the dataset and produce a multiple sequence alignment of the concatenated core genes (present in > 99% strains) using MAFFT. Third, the multiple sequence alignment of the core genome was used to generate a best-fit maximum likelihood phylogeny using IQTREE version 1.6.12 using ModelFinder optimization [32,33]. Finally, the trees were visualized in iTOL version 5.5 [34]. Pangenomes of closely related 16 strains to *L. lactis* N8 were also analyzed by roary_plots.py (https://github.com/sanger-pathogens/Roary/tree/master/contrib/roary_plots; accessed on 5 July 2021) to show the presence and absence of core and accessory genes.

#### 2.3.3. Prophages Prediction

The nucleic acid of the lysogenic phage integrated into the host genome is referred to as prophage. In this study, we examined the prevalence of prophages in *L. lactis* N8 and its derivatives using online prediction tool PHASTER (https://phaster.ca/; accessed on 5 July 2021) [35].

#### 2.3.4. Genomic Islands Prediction

Genomic islands can encode a variety of functions involving symbiosis and pathogenesis. IslandViewer 4 (http://www.pathogenomics.sfu.ca/islandviewer/; accessed on 5 July 2021) was used to predict genomic islands [36].

#### 2.3.5. Genome Mining for Secondary Metabolite Biosynthetic Gene Clusters

Secondary metabolites by bacteria are an important source for identification of novel antimicrobial and bioactive compounds. To excavate this potential of *L. lactis* N8 on top of its excellence in antimicrobial nisin production, secondary metabolite biosynthetic gene clusters of N8 were predicted using the antiSMASH 5.0 (https://antismash.secondarymetabolites.org/; accessed on 5 July 2021) [37]. Swiss-model was used to predict the structure of antimicrobial peptides [38].

### 2.4. Methods of Genome Streamlining

The vectors for the deletion of nonessential fragments were constructed as described in our previous work [12]. Briefly, two fragments of the flanking region of nonessential fragment L1 were amplified by PCR with a proof-reading polymerase (Takara, Dalian, China), and the N8 chromosome was used as template. Then, the fragments were ligated into the *Xho*I–*Swa*I and *Sac*I–*Bgl*II restriction sites of pNZ5319. The recombinant plasmid pNZ5319∆L1 obtained was transformed into *E. coli* DH5α cells strain by CaCl_2_ method [39]. Then, pNZ5319∆L1 was isolated and electroporated into *L. lactis* N8 competent cells to generate mutant *L. lactis* N8-1-*cat*. After the deletion of the L1 fragment, *cat* gene in the mutant was retrieved by introducing the plasmid pNZTS-Cre into *L. lactis* N8-1-*cat* strain. The final mutant was named as *L. lactis* N8-1. Seven nonessential DNA regions were deleted on schedule with the Cre-*loxP* system. The gene knockout vectors pNZ5319∆L2, pNZ5319∆L3 and pNZ5319∆L4, pNZ5319∆L5, pNZ5319∆L6, pNZ5319∆L7 and pNZ5319∆L8 were constructed subsequently. The second nonessential fragment was then deleted in *L. lactis* N8-1 with the plasmid pNZ5319∆L2. The derivative was named *L. lactis* N8-2. The deletion step was repeated with pNZ5319∆L3, pNZ5319∆L4, pNZ5319∆L5, pNZ5319∆L6, pNZ5319∆L7 and pNZ5319∆L8 plasmids. The resulting mutants were named *L. lactis* N8-3, *L. lactis* N8-4, *L. lactis* N8-5, *L. lactis* N8-6, *L. lactis* N8-7 and *L. lactis* N8-8, respectively. The correct deletions of the eight constructed strains were confirmed by resequencing.

### 2.5. Resequencing

Isolation of gDNA was carried out using SDS method. Total DNA obtained was subjected to quality control by agarose gel electrophoresis and quantified by Qubit (Thermo Fisher Scientific, Waltham, MA, USA). The genome of *L. lactis* N8-8 was sequenced with MPS (massively parallel sequencing) Illumina technology (Illumina, San Diego, CA, USA). The DNA library was constructed: a paired-end library with an insert size of 350 bp. The 350-bp library was sequenced using an Illumina PE150 strategy. Library construction and sequencing were performed at the Beijing Novogene Bioinformatics Technology Co., Ltd. Quality control of paired-end reads was performed using in-house program. Then, data processing, reads mapping and SV (structural variation) analysis were conducted as we described previously [12].

### 2.6. Phenotype Testing

#### 2.6.1. Determination of Growth Profiles

To determine the growth profiles, *L. lactis* strains were cultured at 30 °C for 6 h in static (non-aerated) condition to log phase, harvested by centrifugation (5000× *g*, 3 min) and washed twice with PBS (phosphate buffered saline, pH 7.4). After washing, the cells were resuspended (adjusted to the same initial cell concentration) in GM17 medium as seed. Then, the seeds were inoculated into 100 mL GM17 flask culture medium with the ratio of 1% and cultured at 30 °C in static condition; samples were taken every hour for the determination of OD600, and the experiment was repeated three times independently. The maximum growth rate and generation time were calculated by using the data of growth profiles.

#### 2.6.2. Determination of Nisin Titre

Nisin yield was determined by the agar well diffusion method [12] with minor modifications. Briefly, the broth of the tested strains after fermentation was boiled for 10 min and cells were removed by centrifugation at 8000 rpm for 3 min. Then, the supernatant was appropriately diluted with 0.02 M HCl. *M. luteus* (10^7^ CFU/mL) was used as indicator and inoculated at a concentration of 1% (*v/v*) into 30 mL melted/cooled LB agar. To enhance nisin diffusion, 1.5% (*v/v*) Tween 80 (JiangTian, Tianjin, China) was added to the medium and mixed well. Then, the medium was quickly poured into sterile petri dish. After solidification and pre-cultivation, a 7 mm diameter sterile cork borer (MRS Scientific Ltd., Wickford, UK) was used to generate agar well for loading samples. Standard nisin solutions (concentrations of 20, 40, 80, 100, 200 and 400 IU/mL) were prepared using nisin powder (Sigma, St. Louis, MO, USA). Subsequently, standard nisin solutions and sample solutions were, respectively, loaded into the wells (80 μL per well), and the plates were incubated at 37 °C for 24 h. The diameter of inhibition zone was measured with a calliper. A regression equation was derived from the nisin standard data.

### 2.7. Statistical Analysis

The experiments to determine the growth profiles of *L. lactis* were performed in independent biological triplicates, and each sample was additionally collected in technical triplicates. For calculating the maximum growth rate and generation time, the experiments were independently repeated at least three times. Assays to determine the nisin yield of different *L. lactis* strains were independently repeated at least three times. Statistical analyses of data were performed using Origin 85 software version 8.5.0 SRI (OriginLab Corporation, Northampton, MA, USA) and GraphPad Prism 5 software version 5.01 (GraphPad software, Inc, San Diego, CA, USA).

## 3. Results and Discussion

### 3.1. General Features of the Genome

**Figure 1:**
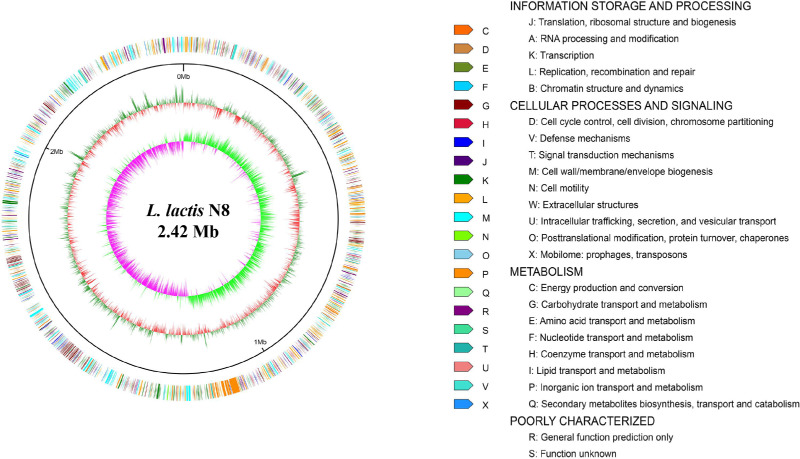
Circular genome plot of *L. lactis* N8. From outer to inner circle: gene function annotation results, genome GC content, genome GC skew.

The assembly of the reads resulted in three contigs, implying that N8 contains one chromosome and two megaplasmids (Figure 1 and Table 2) [21]. N8 has fewer CDs than *L. lactis* KF147 (2446) and *L. lactis* KLDS 4.0325 (2596). The coding regions of N8 have a guanine plus cytosine (GC) content of 35.1 mol%, which is comparable to the other *L. lactis* strains.

### 3.2. Phylogeny and Core-Pan Genome

**Figure 2:**
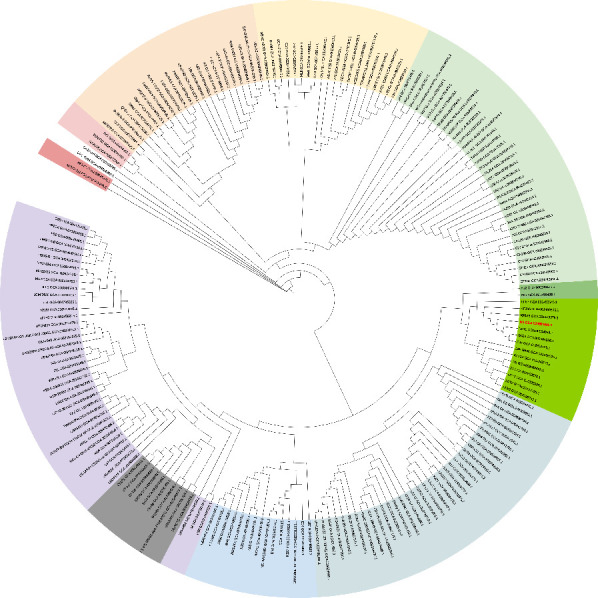
Overall position of *L. lactis* N8 in the *L. lactis* phylogeny. An unrooted tree, representing the phylogenetic position of N8 (red) among 202 *L. lactis*.

To visualize the position of *L. lactis* N8 within the species, we constructed an unrooted phylogenetic tree using all *L. lactis* currently published on NCBI (update on 5 July 2021) (Figure 2), and the rooted phylogenetic tree of all *L. lactis* revealed the distances among the strains, which is shown in Supplementary Figure S1. Fifteen strains are closely related to strain N8 and share the same evolutionary origin, as shown in Figure 2 marked with a green background.

**Figure 3:**
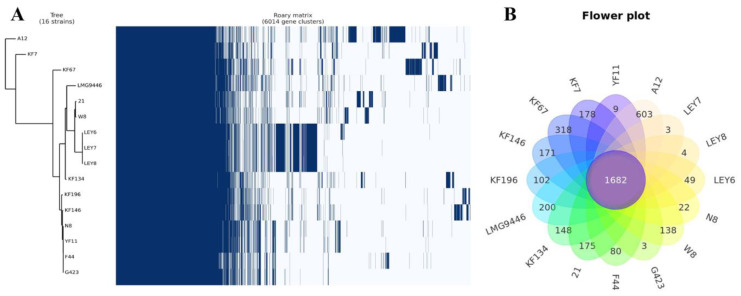
(**A**) Pan-genome matrix of 17 *L. lactis*. Blue, gene presence; white, gene absence. (**B**) Flower plot of 17 *L. lactis*.

The Roary matrix was generated from these 15 strains together with *L. lactis* N8, showing the genetic relatedness among them (Figure 3A). A pan-genome analysis shows that N8 shares the same immediate evolutionary origin with strains YF11, G423 and F44. The closest related *L. lactis* YF11 has a coding density of 95.52% similar to *L. lactis* N8. Meanwhile, the strain N8 has a coding density of 90.51% and 87.50% similar to *L. lactis* G423 and *L. lactis* F44, respectively. From the flower plot in Figure 3B, we can see that these 16 strains have 1682 genes in common, indicating that they are very closely related.

It is worth noting that, previously, *L. lactis* contained four subspecies: *L. lactis* subsp. *lactis*, *L. lactis* subsp. *hordniae*, *L. lactis* subsp. *cremoris* and *L. lactis* subsp. *tructae*. These four subspecies could be divided into two groups based on *recA* sequence analysis: *L. lactis* subsp. *lactis* and *L. lactis* subsp. *hordniae*; *L. lactis* subsp. *cremoris* and *L. lactis* subsp. *tructae*. The two groups had a relatively low DNA–DNA hybridization value (about 60%) [2]. In a recent study, *L. lactis* has been reclassified to three component subspecies, i.e., subsp. *lactis*, subsp. *lactis biovar diacetylactis* and subsp. *hordniae*. The former *L. lactis* subsp. *cremoris* is now an independent species *L. cremoris* sp. nov. and transfer *L. lactis* subsp. *tructae* to *L. cremoris* as *L. cremoris* subsp. *tructae* comb. nov. [40].

### 3.3. Megaplasmids of L. lactis N8

**Figure 4:**
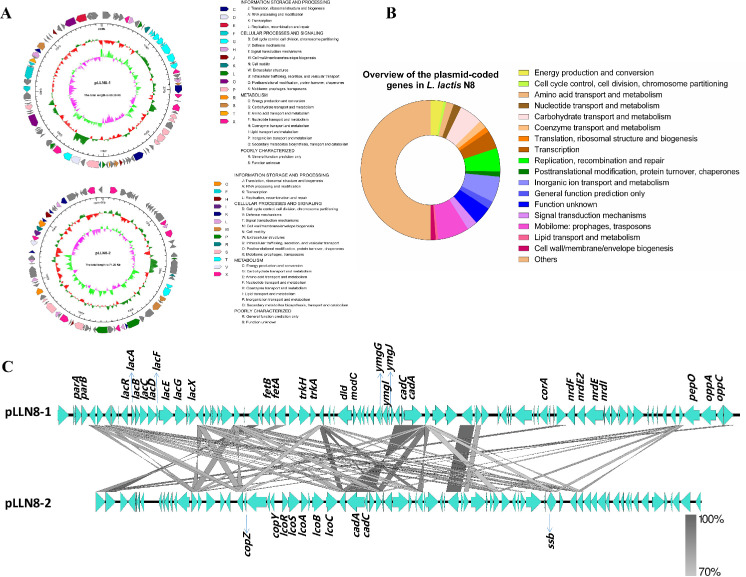
Plasmid genetic maps of *L. lactis* N8. (**A**) Arrows indicate positions, size and direction of predicted genes. Putative functions of genes are presented by colors. From outer to inner circle: gene function annotation results, genome GC content, genome GC skew. (**B**) Overview of the functions of plasmid-encoded genes in *L. lactis* N8. The sum of the sizes of the genes within a category relative to the total size of all plasmid genes was used. (**C**) Comparison of the homology of two large plasmids.

Plasmids, normally dispensable for bacterial growth, are autonomously replicating extrachromosomal DNA entities. They carry backbone genes important for the replication and maintenance in their host and might confer an advantage to their host in its ecological niche [41]. The genome of *L. lactis* N8 was revealed to possess two megaplasmids, designated pLLN8-1 (80,301 bp) and pLLN8-2 (71,261 bp) (Figure 4A). They are stably maintained in their natural host N8 and in total represent 5.89% (151,562 bp) of the N8 genome. An overview of the putative functions of plasmid-encoded genes is shown in Figure 4B. The two megaplasmids carry a number of genes known to be important for growth and survival under a specific environment. These two plasmids carry the genes encoding many functional proteins, for example proteins responsible for protein degradation, bacteriophage biogenesis, stress resistance, mobilization, partition systems, oligopeptide transporter, type I restriction-modification (R-M) system, metal transporters, enzymes and transcriptional regulators. The pLLN8-1 encodes XRE-family HTH domain transcriptional regulator, sugar transport proteins (*lacRABCDFEGX*), cadmium resistance proteins (*cadAC*), potassium transporter (*trkAH*), magnesium transporter (*corA*), putative Asp23/Gls24 family general stress response protein (*ymgGIJ*), oligopeptide transporter (*oppAC*), putative iron export ABC transporter (*fetAB*), chromosome partitioning proteins (*parAB*), ribonucleoside-diphosphate reductase (*nrdFEI*) and several IS3 and IS6 family transposases. Its replication initiator protein is the RepB family protein. The pLLN8-2 encodes functional copper resistance proteins (*lcoRSABC*), copper metabolism proteins (*copYZ*), cadmium resistance proteins (*cadAC*) and several IS3, IS6 and IS982 family transposases. Two highly homologous repB-containing replicons were found in the two plasmids, showing that both plasmids replicate via the theta-type mechanism [3]. Despite their enormous size, both plasmids appeared to be segregationally stable thanks to the possession of partition systems [41]. A detailed comparison of the homology of two large plasmids was shown in Figure 4C.

The plasmid-encoded and well-defined ability to rapidly ferment lactose amongst dairy-associated lactococci is a typical feature of LAB [42]. The *lacABCDFEGX* operon includes genes that function to encode lactose acquisition and utilization and is regulated by the repressor LacR encoded by the divergently oriented *lacR* gene. The Opp proteins belong to a superfamily of highly conserved ATP-binding cassette transporters that mediate the uptake of casein-derived peptides. The *opp* operon is important for the peptide uptake and the production of flavor compounds in food fermentation [3,43]. Together, the plasmid system is of great importance for N8 in terms of sugar acquisition and metabolism, flavor formation and tolerance to multiple environmental stresses.

In addition, we did a horizontal comparison of all *L. lactis* plasmids. At present, 38 strains of *L. lactis* have been announced, carrying 139 natural plasmids, and each strain contains 3.66 plasmids on average. The average size of all the *L. lactis* plasmids is 31,197 bp (Table 3). The average size of the plasmids of N8 is significantly larger than most of strains, indicating that the plasmids of N8 may contain more functional genes. The functional annotations of the two large plasmids can be viewed on the website (https://www.ncbi.nlm.nih.gov/nuccore/NZ_CP059051.1; accessed on 5 July 2021 and https://www.ncbi.nlm.nih.gov/nuccore/NZ_CP059050.1; accessed on 5 July 2021). Interestingly, we found that two large plasmids of N8 contain some unusual fragments of prophage. We postulated that natural plasmids, over a certain size, may have evolved from the prophage. Furthermore, the plasmids contain several IS family transposases that make the plasmid system more like a spare small genome looped from the genome, which can be used to store horizontally transferred fragments.

### 3.4. Prophage-Related Fragments of L. lactis N8

**Figure 5:**
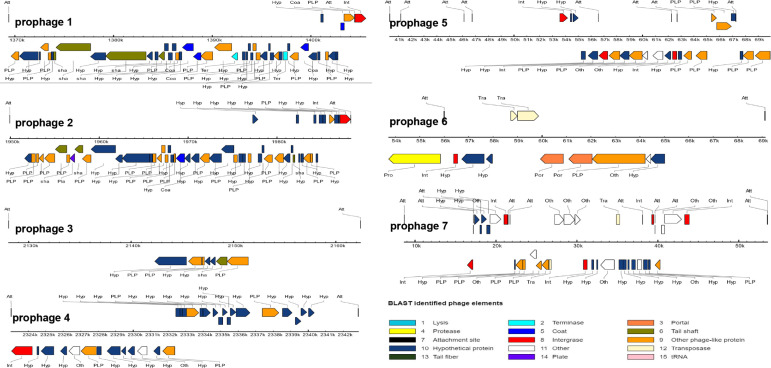
Prediction of the functional protein of each prophage.

The N8 chromosome harbors seven regions that represent prophage-related fragments. Among these seven prophages, LLN8-1 (36,193 bp) and LLN8-2 (38,338 bp) appear to represent intact phages, LLN8-3 (34,367 bp), LLN8-4 (19,856 bp), LLN8-5 (44,895 bp), LLN8-6 (15,398 bp) and LLN8-7 (29,597 bp) appear to represent incomplete phages or questionable phages. The BLAST results of each prophage-related fragment are shown in Figure 5. Together, bacteriophage sequences encompass approximately 8.98%, representing a large portion of the N8 genome. The seven prophage-related fragments occupy various positions on the N8 genome. The G+C content of seven prophages ranges from 32.05 mol% to 36.09 mol%, similar to the value of 35.1 mol% calculated for the N8 chromosome (Table 4).

We have performed prophage predictions on all 42 *L. lactis* strains with complete genomes currently published by NCBI, and the results were listed in Supplementary Table S4. We found that all the published strains with complete genomes contain prophages to different degrees (intact, incomplete or questionable), indicating the symbiotic relationship between phages and *L. lactis* during the long-term evolution. Bacteriophages play a very important role in the evolution of lactic acid bacteria. There are 145 intact prophages identified from 42 *L. lactis* genomes (Supplementary Table S4). Among them, only five *L. lactis* strains carried no intact prophage; seventeen strains carried five or more intact prophages. On average, each strain has 6.5 prophage-related fragments.

Currently, 42 complete and 160 partially assembled *L. lactis* genomes are available in NCBI’s GenBank (update on 15 July 2021). The intact bacteriophages or prophage remnants have been identified in each one [44,45,46]. Possibly due to their long history in milk fermentation, *L. lactis* are regarded to contain the highest number of prophages among the LAB [47]. Prophage-related fragments encompass from 3 to 10% of the total genome of *L. lactis* strains [46]. The genome sequence analyses of *L. lactis* prophages indicated that they are affiliated with the P335 group of phages, and the two other species (c2- and 936-like) are composed of phages that are exclusively virulent [48]. In N8, LLN8-1, LLN8-2, LLN8-3, LLN8-4, LLN8-5, LLN8-6 and LLN8-7 have the highest homology with the staphylococcal phage phi3396, lactococcal phage TP901-1, *Gordonia* phage *Hotorobo*, lactococcal phage bIL310, staphylococcal bacteriophage SPbeta-like, staphylococcal bacteriophage SPbeta-like and clostridium phage phiCD6356, respectively.

### 3.5. Genomic Islands of L. lactis N8

The genomic islands (GIs) are large DNA segments generally between 10 and 200 kb in length, with special structure and function. GIs have a variety of biological functions, such as antibiotic resistance, pathogenicity, xenobiotic degradation and heavy metal resistance [49,50]. The common features of GIs include: (1) integration hotspots for GIs are usually adjacent to RNA genes on chromosome; (2) GIs are flanked by direct repeats that are possibly related to the horizontal transfer of GIs; (3) horizontal transfer related genes, such as transposases, integrases and recombinases, are often found at the junction of GIs and core genome; (4) there are significant differences in G+C content between GIs and core genome [51].

**Figure 6:**
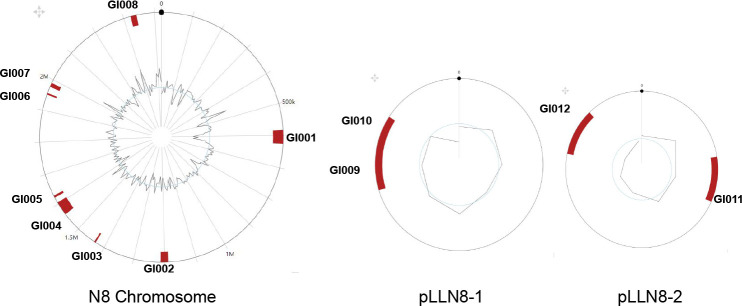
Circular map of *L. lactis* N8 chromosome and two large plasmids showing the physical location of the 12 GIs.

In this study, twelve GIs in N8 accounting for 4.12% of the total genome were predicted, and their physical locations on the genome were marked in Figure 6. The detailed information of the GIs is shown in Table 5. Detailed gene content was listed in Supplementary Table S5. The prophage fragments and GIs overlap to a certain extent. This may be due to their exogenous DNA nature, obtained through horizontal transfer or other means of genomic evolution [52]. Owing to the diversity of the sources of GIs, their G+C content is different from the N8 genome.

### 3.6. Other Characteristics of L. lactis N8

#### 3.6.1. Cell-Surface Protein

In *L. lactis*, *csc* genes can be found both on the chromosome and on the plasmids [53]. The chromosomally located *csc* loci are commonly flanked by IS elements and are, therefore, thought to be horizontally acquired and mobile. A *csc* gene cluster encoding exclusively cell-surface proteins was identified in N8. The gene cluster generally has one copy of four new gene families called *cscA*, *cscB*, *cscC* and *cscD* [53]. All the encoded proteins have a signal peptide for secretion by the secdependent pathway, while some have cell-surface anchors, novel WxL domains and putative domains for sugar binding and degradation. A proteomic analysis on N8 shows that the *cscA-D* genes are co-expressed [12], supporting their operon organization. Researchers propose that the CscA, CscB, CscC and CscD proteins form cell-surface protein complexes and play a role in carbon source acquisition [53]. Their presence in dairy lactococci is perhaps a relic of their plant ancestral heritage, although they may still provide some unknown benefits to their host in the dairy environment [3].

#### 3.6.2. PKS/NRPS

**Figure 7:**
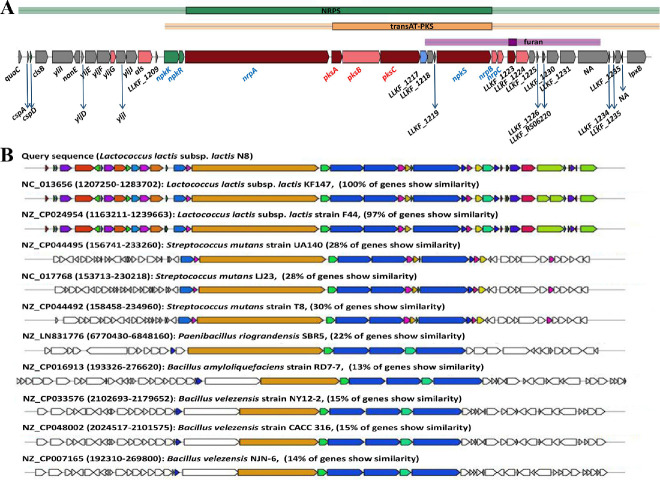
(**A**) The hybrid NRPS/PKS from *L. lactis* N8. Genes on the hybrid NRPS/PKS system are colored as follows: two-component transcriptional regulator (green), secondary metabolite core biosynthetic genes (dark red), additional biosynthetic genes (light red), transport-related genes (blue) and other genes (grey). (**B**) The homology of genes in ten other strains of *L. lactis*, *S. mutans*, *P. riograndensis*, *B. amyloliquefaciens and B. velezensis* (NCBI Reference Sequence numbers are written next to the strain names).

*L. lactis* KF147 is the first reported *L. lactis* that has identified a gene cluster encoding a hybrid non-ribosomal peptide synthetase and polyketide synthase (NRPS–PKS) system [54]. Here, we reported that N8 also harbors the NRPS–PKS system (Figure 7A). Currently, PKS can be roughly divided into three types, namely PKS I (modular), PKS II (iterative) and PKS III (chalcone). The diverse activities of the PKS are completed by their protein modules, and the protein modules from different sources may be different. As a result, the chemical structure of polyketone compounds is also different. We compared the secondary metabolite systems of 42 *L. lactis* strains. We found that almost all the *L. lactis* contain the type Ⅲ PKS gene cluster. However, only eight contained NRPS gene clusters, namely *L. lactis* N8, *L. lactis* F44, *L. lactis* KF196, *L. lactis* KF147, *L. lactis* G423, *L. lactis* NCDO2118, *L. lactis* YF11, *L. lactis* KLDS 4.0325, all of which but *L. lactis* KLDS 4.0325 could produce lanthiopeptide (Supplementary Table S6).

The homology of the genes of the NRPS/PKS system in ten other strains was compared with N8, and the results are shown in Figure 7B. In the hybrid NRPS–PKS system of *L. lactis* N8, six NRPS modules and three PKS modules were identified (Figure 7A). Oxidative stress resistance and biofilm formation are the most probable functions of this hybrid system [55].

#### 3.6.3. Bacteriocin Gene Cluster

There are two bacteriocin gene clusters in the genome of N8. The first one is the well-known nisin gene cluster encoding nisin synthesis-related protein. The second bacteriocin cluster in N8 consists of nine genes, including *yujA*, *yujB*, *yujD*, *yujE1*, *yujE2*, *yujF*, *yujG* and two genes of unknown function. Among them, *yujG* encodes a LytTR family transcriptional regulator YujG, and *yujF* encodes bacteriocin precursor peptide YujF, while *yujD*, *yujE1* and *yujE2* encode putative immunity proteins YujD, YujE1 and YujE2, respectively. Furthermore, *yujB* encodes uncharacterized integral membrane protein YujB, and *yujA* encodes class I SAM-dependent methyltransferase YujA, which catalyzes the methylation of one or more specific substrates using S-adenosyl-L-methionine (SAM or AdoMet) as the methyl donor. The remaining two genes encode membrane GTPase and hypothetical protein. The Swiss Model prediction results show that there are conserved regions between YujF and lactococcin 972. In addition, the prediction results indicate that YujF may have secretion signal peptide (Supplementary Figure S2).

### 3.7. Construction of Streamlined Genome Chassis of L. lactis N8

**Figure 8:**
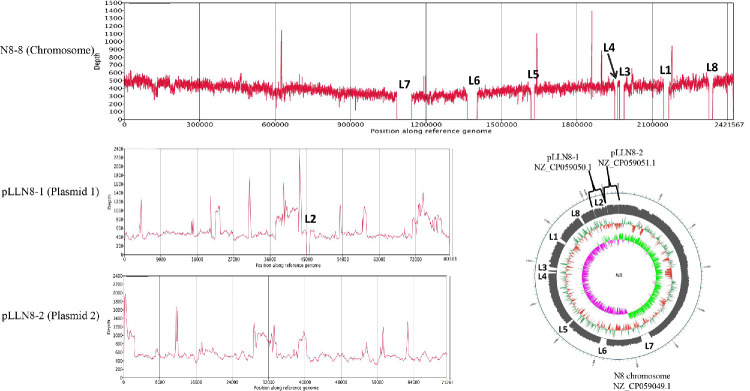
Alignment of *L. lactis* N8 and *L. lactis* N8-8 genome sequences verified correct deletion.

Through genome-wide analysis, we found prophages and GIs that are nonessential fragments for N8. These nonessential fragments were our preferred deletion targets for constructing the N8 streamlined genome chassis. In this study, we successfully deleted eight segments and realized the current maximum genome simplification of *L. lactis* (Figure 8). The cumulative deletion was 176.43 kb, which accounts for 6.86% of the whole N8 genome (Table 6). We verified the correctness of the knockout of the fragments through resequencing technology, and the physical location of each deleted fragment was also marked (Figure 8).

**Figure 9:**
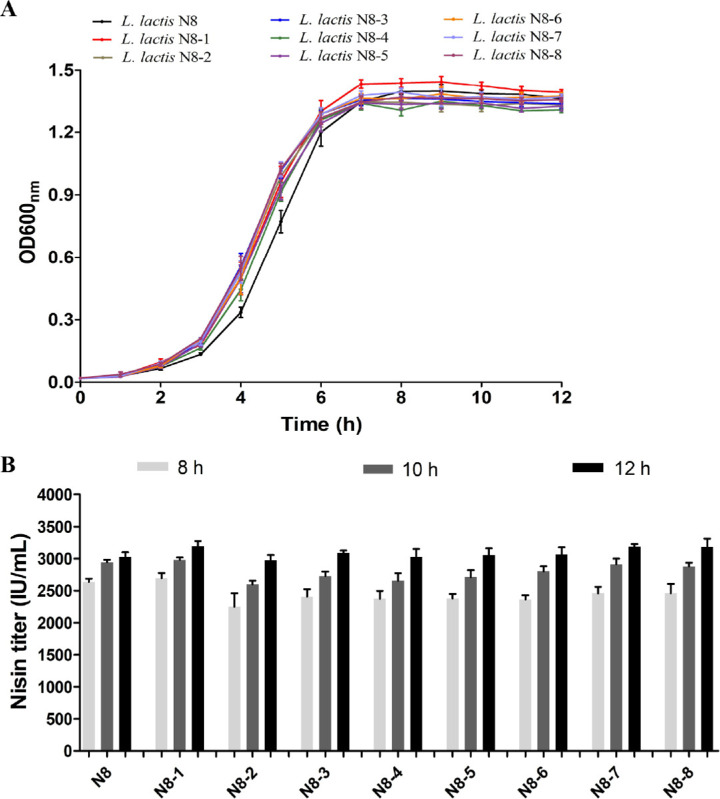
Phenotypes of wild-type strain and streamlined genome chassis strains. (**A**) Growth profiles s of the strains. (**B**) Nisin titer of different strains at different time points (8 h, 10 h and 12 h).

Then, we tested the growth phenotype of all the genome-streamlined strains. The growth status of the simplified genome strains is not much different from that of the wild-type strain N8 (Figure 9A). The final OD600 value is not significantly different, indicating that the deletion of these fragments did not cause a growth defect to the strains. However, it can be seen from the growth period that the deletion strains reach the stable period earlier than the wild type. Through the calculation of the generation time, we found that the generation times of all the deleted strains are shorter than that of the wild-type strain (Table 6). Especially, the generation time of strain N8-8 with the largest amount of deletion was shortened by 17.18% compared with the wild-type strain. Compared with the wild-type strain, the generation times of N8-3, N8-4 and N8-7 were shortened by 12.29%, 14.25% and 16.79%, respectively. Except for N8-5, the maximum growth rate of the deleted strains is also higher than that of the wild-type strains, which proves that deleting nonessential genome fragments is feasible for reducing the physiological burden of the strains (Table 6).

Due to the high nisin yield of N8, another phenotype we are more concerned about is how the deletion of nonessential fragments affects the yield of nisin. Therefore, we tracked the nisin production of all the strains (wild-type and genome-streamlined strains). As shown in Figure 9B, the nisin titre of N8-2 was slightly lower than that of the wild-type strain after 8 h of cultivation, but the difference in the nisin yield among all the strains disappeared after 12 h of cultivation. It shows that the deletion of nonessential fragments did not affect the overall nisin production, which is in line with our expectations. In the future, we will use the chassis strains to design a metabolic module for further increasing the production of nisin. In addition, the chassis strain N8-8 has the possibility of further genomic deletions, for example, at the area of remaining prophages and GIs. This strain possesses a great potential for the development of a model lactic acid bacterial strain. The construction of a simplified chassis strain of *L. lactis* is also important for understanding the minimal genome of its life-sustaining activities. In addition, the construction of simplified genome chassis strains also laid the foundation for the application of *L. lactis* in the field of synthetic biology.

## 4. Conclusions

In this study, we reported detailed genome features of *L. lactis* N8. Through the phylogenetic tree and core-pan genome analysis, we found that *L. lactis* N8 has the highest homology with *L. lactis* YF11. Through a genome-wide analysis, we found that N8 has two large plasmids harboring many functional genes. These two large plasmids play a very important role in N8 sugar transport and metabolism, flavor formation and stress tolerance under special environmental conditions. Then, we counted all the plasmids of *L. lactis* currently published and found that the plasmids possessed by N8 are significantly larger than most of other *L. lactis*. In addition, we predicted the prophage in N8 and counted all the prophages of *L. lactis* with the complete genome published so far. We also predicted the GIs in N8 and marked their precise locations. Based on these genome-wide findings, we set out to construct a streamlined chassis strain from *L. lactis* N8. Prophage and genomic island fragments were the preferred deletion targets, and we successfully deleted 6.86% of the N8 genome. As far as we know, we are the first to achieve this large an amount of deletion of an *L. lactis* genome, which is of great significance for the study of the simplest functional genome of lactic acid bacteria. The simplification of the *L. lactis* genome can also expand the application of lactic acid bacteria in the field of synthetic biology by, e.g., producing favorable metabolic modules in the *L. lactis* chassis strain.

## Figures and Tables

**Table 1:** Bacterial strains and plasmids utilized in this study.

Strains or Plasmids	Relevant Descriptions	Reference
Strains		
*E. coli* DH5α	Cloning host; F-*φ80lacZ∆M15endA1 recA1 endA1 hsdR17* (rK-mK+) *supE44 thi-1 gyrA 96 relA1 ∆(lacZYA-argF)U169 deoR λ*-	[22]
*E. coli* DH5α-up-down (L1)	Cm^r^, Em^r^, *E. coli* DH5α derivative containing the whole plasmid pNZ5319∆L1	[12]
*E. coli* DH5α-up-down (L2)	Cm^r^, Em^r^, *E. coli* DH5α derivative containing the whole plasmid pNZ5319∆L2	This study
*E. coli* DH5α-up-down (L3)	Cm^r^, Em^r^, *E. coli* DH5α derivative containing the whole plasmid pNZ5319∆L3	This study
*E. coli* DH5α-up-down (L4)	Cm^r^, Em^r^, *E. coli* DH5α derivative containing the whole plasmid pNZ5319∆L4	This study
*E. coli* DH5α-up-down (L5)	Cm^r^, Em^r^, *E. coli* DH5α derivative containing the whole plasmid pNZ5319∆L5	This study
*E. coli* DH5α-up-down (L6)	Cm^r^, Em^r^, *E. coli* DH5α derivative containing the whole plasmid pNZ5319∆L6	This study
*E. coli* DH5α-up-down (L7)	Cm^r^, Em^r^, *E. coli* DH5α derivative containing the whole plasmid pNZ5319∆L7	This study
*E. coli* DH5α-up-down (L8)	Cm^r^, Em^r^, *E. coli* DH5α derivative containing the whole plasmid pNZ5319∆L8	This study
*Micrococcus luteus* NCIB 8166	Indicator strains for Nisin agar gel diffusion assay	[23]
*L. lactis* N8	Wild-type (WT) Nisin Z producer	[24]
*L. lactis* N8-1	The first DNA region L1 deletion in *L. lactis* N8	[12]
*L. lactis* N8-2	The L2 deletion in *L. lactis* N8-1	This study
*L. lactis* N8-3	The L3 deletion in *L. lactis* N8-2	This study
*L. lactis* N8-4	The L4 deletion in *L. lactis* N8-3	This study
*L. lactis* N8-5	The L5 deletion in *L. lactis* N8-4	This study
*L. lactis* N8-6	The L6 deletion in *L. lactis* N8-5	This study
*L. lactis* N8-7	The L7 deletion in *L. lactis* N8-6	This study
*L. lactis* N8-8	The L8 deletion in *L. lactis* N8-7	This study
Plasmids		
pNZ5319	Cm^r^, Em^r^, used as knock-out vector	[25]
pNZTS-Cre	Em^r^, cre gene cloned at the EcoRI and HindIII sites (cat gene deletion vector)	[20]
pNZ5319∆L1	Cm^r^, Em^r^, upstream and downstream homology arm of L1 amplified from *L. lactis* N8 genome cloned into pNZ5319	[12]
pNZ5319∆L2	Cm^r^, Em^r^, upstream and downstream homology arm of L2 amplified from *L. lactis* N8 genome cloned into pNZ5319	This study
pNZ5319∆L3	Cm^r^, Em^r^, upstream and downstream homology arm of L3 amplified from *L. lactis* N8 genome cloned into pNZ5319	This study
pNZ5319∆L4	Cm^r^, Em^r^, upstream and downstream homology arm of L4 amplified from *L. lactis* N8 genome cloned into pNZ5319	This study
pNZ5319∆L5	Cm^r^, Em^r^, upstream and downstream homology arm of L5 amplified from *L. lactis* N8 genome cloned into pNZ5319	This study
pNZ5319∆L6	Cm^r^, Em^r^, upstream and downstream homology arm of L6 amplified from *L. lactis* N8 genome cloned into pNZ5319	This study
pNZ5319∆L7	Cm^r^, Em^r^, upstream and downstream homology arm of L7 amplified from *L. lactis* N8 genome cloned into pNZ5319	This study
pNZ5319∆L8	Cm^r^, Em^r^, upstream and downstream homology arm of L8 amplified from *L. lactis* N8 genome cloned into pNZ5319	This study

**Table 2:** Basic genomic characteristics of *L. lactis* N8.

Feature	Size/Percentage/Number
Genome size (bp)	2,421,567
G + C content	35.1%
rRNAs	19
tRNAs	64
ncRNAs	4
Total genes	2521
Coding sequences (CDs)	2434
Prophages	7
Genomic islands (GIs)	12
Plasmids	2

**Table 3:** Horizontal comparison of all *L. lactis* plasmids.

Organism Name	Strain	BioSample	Number of Plasmids	Total Size of Plasmids	Release Date
*Lactococcus lactis* subsp. *lactis*	N8	SAMN15500618	2	0.151562	22 October 2020
*Lactococcus lactis* subsp. *lactis*	No attributes	SAMN14223931	1	0.060232	10 September 1998
*Lactococcus lactis* subsp. *lactis*	CBA3619	SAMN11843663	1	0.107586	16 August 2019
*Lactococcus lactis* subsp. *lactis*	FDAARGOS_865	SAMN13450395	1	0.058335	15 December 2020
*Lactococcus lactis* subsp. *lactis*	FDAARGOS_887	SAMN13450417	4	0.180362	15 December 2020
*Lactococcus lactis* subsp. *lactis*	FDAARGOS_866	SAMN13450396	4	0.241108	15 December 2020
*Lactococcus lactis* subsp. *lactis*	FDAARGOS_1064	SAMN16357233	4	0.112979	21 December 2020
*Lactococcus lactis* subsp. *lactis*	WiKim0098	SAMN16788728	1	0.076987	18 January 2021
*Lactococcus lactis* subsp. *lactis*	A12	SAMEA4005236	4	0.126727	23 July 2016
*Lactococcus lactis* subsp. *lactis*	229	SAMN04955249	5	0.165685	5 April 2017
*Lactococcus lactis* subsp. *lactis*	275	SAMN04955252	4	0.25801	5 April 2017
*Lactococcus lactis* subsp. *lactis*	UC063	SAMN04956302	5	0.149078	5 April 2017
*Lactococcus lactis* subsp. *lactis*	UL8	SAMN04956402	3	0.037067	10 April 2017
*Lactococcus lactis* subsp. *lactis*	14B4	SAMN08792430	1	0.0597	1 June 2018
*Lactococcus lactis* subsp. *lactis*	184	SAMN04955247	6	0.042416	11 January 2019
*Lactococcus lactis* subsp. *lactis*	C10	SAMN04956267	3	0.056865	11 January 2019
*Lactococcus lactis* subsp. *lactis*	UC06	SAMN04956292	6	0.155687	13 January 2019
*Lactococcus lactis* subsp. *lactis*	UC08	SAMN04956293	5	0.166766	16 January 2019
*Lactococcus lactis* subsp. *lactis*	UC77	SAMN04956303	5	0.178184	3 January 2019
*Lactococcus lactis* subsp. *lactis*	UC11	SAMN04956294	6	0.156382	11 September 2019
*Lactococcus lactis* subsp. *lactis*	G121	SAMN14943687	3	0.123307	7 September 2020
*Lactococcus lactis* subsp. *lactis*	223	SAMN09847869	6	0.05587	25 February 2021
*Lactococcus lactis* subsp. *lactis*	WM1	SAMN09847649	5	0.180672	23 February 2021
*Lactococcus lactis* subsp. *lactis*	267	SAMN09847870	5	0.164327	27 February 2021
*Lactococcus lactis* subsp. *lactis*	DRC3	SAMN16604567	7	0.232822	10 April 2021
*Lactococcus lactis* subsp. *lactis*	Lac3	SAMN18740314	1	0.007367	13 May 2021
*Lactococcus lactis* subsp. *lactis bv. diacetylactis*	FM03	SAMN06061939	7	0.0809	24 May 2017
*Lactococcus lactis* subsp. *lactis bv. diacetylactis*	SD96	SAMN12502795	10	0.219931	7 October 2019
*Lactococcus lactis* subsp. *lactis bv. diacetylactis*	BGBU1-4	SAMN12627231	1	0.00633	17 November 2019
*Lactococcus lactis* subsp. *lactis bv. diacetylactis*	S50	SAMN10167144	6	0.240412	10 September 2020
*Lactococcus lactis* subsp. *lactis bv. diacetylactis*	No attributes	SAMN14226089	1	0.018977	1 August 2011
*Lactococcus lactis* subsp. *lactis bv. diacetylactis*	No attributes	SAMN14226088	1	0.021728	1 August 2011
*Lactococcus lactis* subsp. *lactis bv. diacetylactis*	No attributes	SAMN14226087	1	0.022166	1 August 2011
*Lactococcus lactis* subsp. *lactis bv. diacetylactis*	No attributes	SAMN14226080	1	0.053876	1 August 2011
*Lactococcus lactis* subsp. *lactis*	CV56	SAMN02603398	5	0.119279	1 May 2012
*Lactococcus lactis* subsp. *lactis*	KF147	SAMN02603087	1	0.03751	22 December 2009
*Lactococcus lactis* subsp. *lactis*	KLDS 4.0325	SAMN02603468	6	0.171622	10 May 2018
*Lactococcus lactis* subsp. *lactis*	NCDO 2118	SAMN02471376	1	0.037571	21 January 2015
Average			3.66	0.031197	

**Table 4:** Information of prophage-related fragments.

Prophage	Start	End	Size (bp)	Att Core Sequence	G+C Content	Status
LLN8-1 (chromosome)	1,369,374	1,405,566	36,193	TTTAATTTAGAAA	35.27%	intact
LLN8-2 (chromosome)	1,949,930	1,988,317	38,388	AACGTAACTAAAAACGTAACTAA	35.18%	intact
LLN8-3 (chromosome)	2,128,037	2,162,403	34,367	AACTTATTTTTAT	34.31%	incomplete
LLN8-4 (chromosome)	2,323,015	2,342,870	19,856	ACGCTTTTTACTACGTTCG	34.56%	incomplete
LLN8-5 (plasmid1)	40,333	69,929	29,597	AAAATAAAAAGT	32.05%	incomplete
LLN8-6 (plasmid2)	8658	53,552	44,895	TTTCGAACATTT	36.09%	questionable
LLN8-7 (plasmid2)	53,728	69,125	15,398	AGGTTCTGTTGCAAAGTT	35.32%	questionable

**Table 5:** Characterization of genomic islands in *L. lactis* N8.

Genomic Island	Start	End	Size (bp)	G+C Content %
GI001 (chromosome)	583,454	626,486	43,032	30.63
GI002 (chromosome)	1,188,697	1,213,410	24,713	32.54
GI003 (chromosome)	1,431,036	1,435,913	4877	39.57
GI004 (chromosome)	1,560,579	1,604,865	44,286	35.98
GI005 (chromosome)	1,622,309	1,629,361	7052	30.91
GI006 (chromosome)	1,950,028	1,955,065	5037	30.06
GI007 (chromosome)	1,976,159	1,989,281	13,122	33.38
GI008 (chromosome)	2,322,308	2,341,772	19,464	34.55
GI009 (plasmid 1)	56,384	60,867	4483	37.20
GI010 (plasmid 1)	58,996	67,736	8740	33.58
GI011 (plasmid 2)	15,929	22,625	6696	36.81
GI012 (plasmid 2)	55,774	62,660	6886	32.64

**Table 6:** Growth parameters in shaken-flask cultures of *L. lactis* strains.

*L. lactis*	Removed (bp)	Cumulative (bp)	Deletion (%)	μmax (h^−1^)	Generation Time (min)
N8	0	0	0%	0.44 ± 0.04	45.7 ± 2.7
N8-1	19,739	19,739	0.77%	0.48 ± 0.05	41.5 ± 5.0
N8-2	1638	21,377	0.83%	0.49 ± 0.06	42.7 ± 4.1
N8-3	18,628	40,005	1.55%	0.50 ± 0.03	40.7 ± 5.0
N8-4	10,659	50,664	1.97%	0.47 ± 0.03	40.0 ± 5.1
N8-5	13,502	64,166	2.49%	0.44 ± 0.02	41.3 ± 4.9
N8-6	39,203	103,369	4.02%	0.53 ± 0.09	41.1 ± 3.2
N8-7	58,594	161,963	6.29%	0.52 ± 0.03	39.3 ± 5.2
N8-8	14,465	176,428	6.86%	0.50 ± 0.05	39.0 ± 4.2

## Data Availability

Data are contained within the article or Supplementary Materials.

## Supplementary Materials

The following are available online at https://www.mdpi.com/article/10.3390/microorganisms10010047/s1, Supplementary Figure S1. The rooted phylogenetic tree of all 202 *L. lactis*. Supplementary Figure S2. Swiss Model prediction results of YujF. Supplementary Table S1. Primers used in this study. Supplementary Table S2. All 202 *L. lactis* data retrieved by latest 5th July 2021. Supplementary Table S3. Script for converting GFF files to GFF3 files. Supplementary Table S4. Prophage prediction results on 42 *L. lactis* strains with complete genomes currently published by NCBI. Supplementary Table S5. The detailed information of 12 GIs. Supplementary Table S6. Secondary metabolite biosynthetic gene cluster prediction results of 43 *L. lactis* strains (including *L. lactis* N8) with complete genomes currently published by NCBI.
