# Supplementary figures and images for: Genomic Features and Construction of Streamlined Genome Chassis of Nisin Z Producer Lactococcus lactis N8
Source: Microorganisms. 2021 Dec 27;10(1):47. doi: 10.3390/microorganisms10010047 (PMC8779420; doi:10.3390/microorganisms10010047)

Tree scale: 0.01

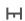

Supplementary Figure S1. The rooted phylogenetic tree of all 202 *L. lactis*.

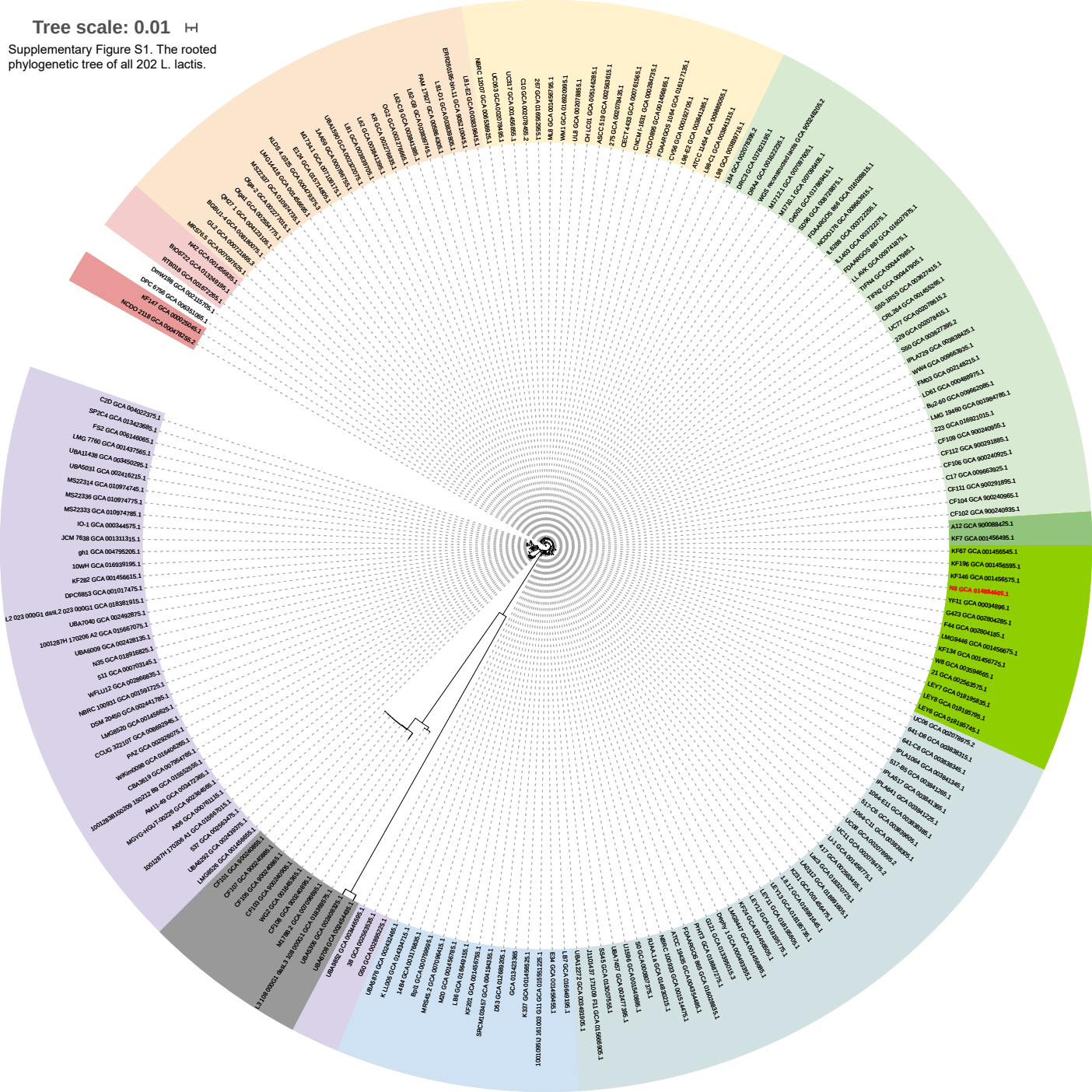

Supplement: Supplementary file 1 [file microorganisms-10-00047-s001.zip › Supplementary Figure S1.pdf]

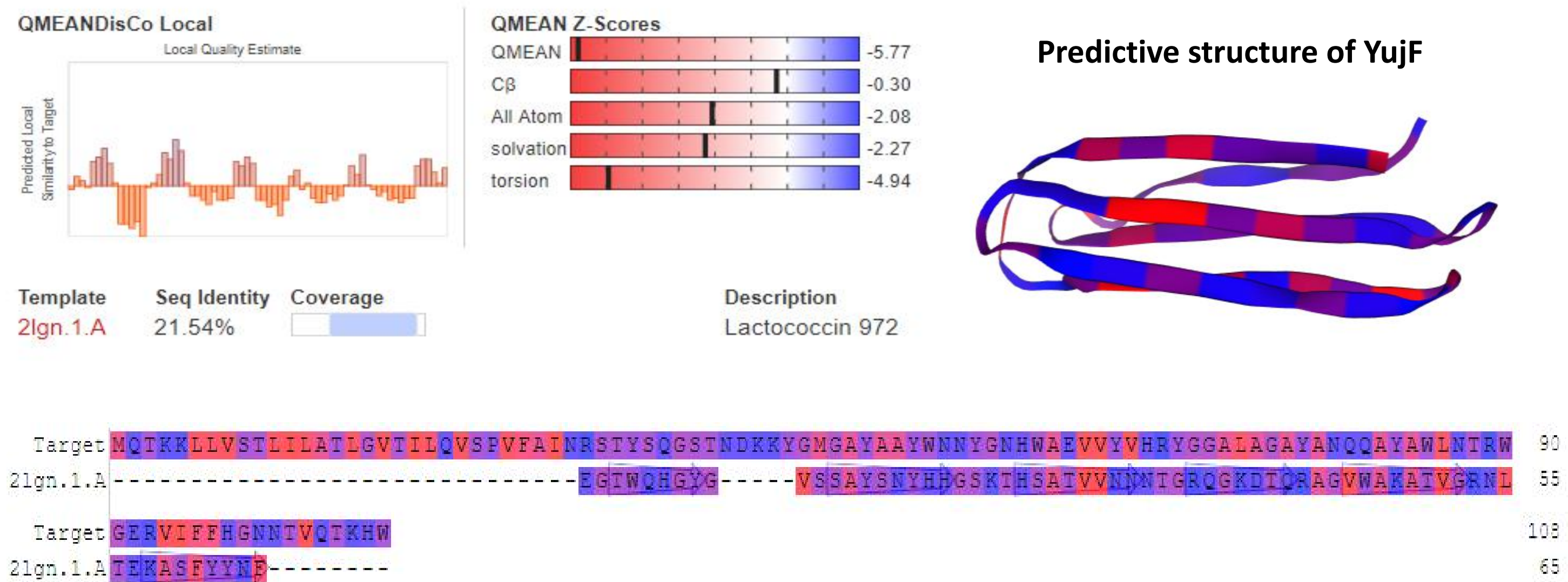

Supplementary Figure S2. Swiss Model prediction results of YujF.

Supplement: Supplementary file 1 [file microorganisms-10-00047-s001.zip › Supplementary Figure S2.pdf]
